# 

**Published:** 1851-07

**Authors:** 


### CONTENTS OF VOLUME IV.

Address of Dr. James Taylor, on filling Teeth, - - - - 1, 53, 105
Artificial Teeth on the atmospheric principle, - - - - - - 35
American Society of Dental Surgeons, Ed - - - - - 50
Atmospheric Pressure, by James Fleming, M. D., - - - - - - 94
Atmospheric pressure plates, Ed. - - - - - - - - - 101
Alcock's Teeth, Ed. - - - - - - - - - - - 153
A test of Levett's Patent Enamel, - - - - - - - - - 189
Agents for the Register, Ed. - - - - - - - - - - - 207
American Medical Association and Dental Colleges, Ed. - - - - 201
Back numbers of the Register, - - - - - - - - - - 51
Bond's Dental Medicine, Ed. - - - - - - - - - - 155
Committee of examination, Ed. - - - - - - - - - 50
Corundrum wheels, Ed. - - - - - - - - - - - 104
Contributions to the Library and Museum, O. C. D. S., Ed., - - - 203
Dentition, by Dr. Taft, - - - - - - - - - 21
Dental Removal, - - - - - - - - - 50
Diseases and Surgical operations of mouth &c. &c.. Ed. - - - 104
Denhard's Teeth, Ed. - - - - - - - - - 154
Dunlevy's Gold Foil, Ed.. - - - - - - - - 205
Dental Lathe, Ed., - - - - - - - - - 206
Editorial Correspondence, - - - - - - - - - 97, 197
Errata, - - - - - - - - - - - - 104
Ether and Chloroform in Surgery, - - - - - - - 155
Flagg's Lateral cavity plates. - - - - - - - 31
Fire, Ed., - - - - - - - - - - - - 104
Fusible Cement, Ed., - - - - - - - - - 152
Food and the Teeth, by James Paul, M. D., - - - - - - - 161
Filling the Teeth after the Lining Membrane has been exposed, Ed., - 204
Gilbert's Patent Cavity Plate, - - - - - - - 33
Improvement in the method of mounting Artificial Teeth, - - - 38
Jones, White & Co's Premium Teeth, Ed.. - - - - - 153
Letter from Dr. Sanford, - - - - - - - - 82
Medical College of Ohio, Ed., - - - - - - - - 52
My Last Amalgam Filling, - - - - - - - - 116
Mastication, by Dr. E. W. Power, - - - - - - - 122
Morton's Teeth, Ed., - - - - - - - - - - 153
Medical students guide in extracting Teeth, Ed,, - - - - - 156
Ninth annual meeting American S. D. S., - - - - - - 40, 84, 125
Neurology, by Dr. E. Buckwell, - - - - - - - - 157
Ohio College Dental Surgery, Ed. - - - - - - - 155
Observations on Exostosis, by D. Vandenburgh, - - - - 194
Ossification of the Pulp of the Teeth, - - - - - - 198
Partial sets of Teeth, by A Perry, D. D. S., - - - - - - 114
Pivot Teeth, by J. Taft, D. D. S., - - - - - - - 119
Porcelain Teeth, Ed. - - - - - - - - - - 153
Sixth annual meeting M. V. A. D. S., - - - - - - - 28
Stockton's Teeth, Ed., - - - - - - - - - - 153
Salivary Calculus, by F. Y. Clark, S. D., - - - - - - - 187
Subscribers to the Register, - - - - - - - - - - 207
Transylvania School of Dental Surgery, Ed., - - - - - - 104
To manufacturers of Porcelain Teeth, Ed., - - - - - - 154
Whole sets of Teeth, by W. E. Ide, D. D. S. - - - - - - - 25, 77

